# A Novel Restraining Device for Small Animal Imaging Exams: Validation in Rabbits

**DOI:** 10.1155/2015/571729

**Published:** 2015-05-31

**Authors:** Carlos Henrique Barbosa, Antonio Carlos Carvalho, Sérgio de Souza, Fernanda Machado, Fábio Guedes, André Monteiro, Alberto Schanaider

**Affiliations:** ^1^Center of Experimental Surgery, Department of Surgery, School of Medicine, Federal University of Rio de Janeiro, 21941-913 Rio de Janeiro, RJ, Brazil; ^2^Orofacial Pain and Masticatory Dysfunction Clinic, Department of Dental Clinic, School of Dentistry, Federal University of Rio de Janeiro, 21941-902 Rio de Janeiro, RJ, Brazil; ^3^Department of Radiology, School of Medicine, Federal University of Rio de Janeiro, 21941-913 Rio de Janeiro, RJ, Brazil; ^4^Department of Pathology and Oral Diagnosis, Dental School, Federal University of Rio de Janeiro, 21941-902 Rio de Janeiro, RJ, Brazil

## Abstract

*Objective*. To develop, validate, and patent a Restraining Device for Small Animal Imaging Exams (RDSAIE) that allows exams to be comfortably conducted without risks to animals and professionals. *Methods*. A RDSAIE with a mobile cover and shelf was built with transparent acrylic material. A total of six anesthetized rabbits were used to perform the following imaging exams of the skull: Cone Beam Computed Tomography, Magnetic Resonance Imaging, and Scintigraphy. *Results*. The device showed great functionality and full visibility of the animal behavior, which remained fully stabilized and immobilized in either the horizontal or vertical position without the need for a person to remain in the test room to assist them. The procedures were performed without difficulty, and images of good resolution and without artifacts were obtained. *Conclusion*. The RDSAIE is comfortable, safe, efficient, and ergonomic. It allows the easy placement of animals in different body positions, including the vertical, the maintenance of postural stability, and full visibility. It may be constructed for animals heavier than 4 kg and it is adaptable for translational studies in *anima nobile*.

## 1. Introduction

Proper restraint and immobilization with comfort and safety during imaging exams are challenges faced by professionals in the field of small animal experimental research. Usually, animals are maintained in transfer boxes until they reach the location where the examination will be performed. Several research institutions, specialized clinics, and veterinary hospitals do not have facilities with proper equipment to perform imaging exams in small animals. The available devices consist of a surgical board with restraints for the limbs of smaller dogs and rodents or guillotine-type boxes for rabbits that need to be altered to visualize the effects of anesthesia. In the testing room, the anesthetized animals are placed on top of plastic equipment or pads without proper restraint. If the anesthesia becomes superficial, imaging may become impaired, the animals can be injured by falling from the device, and/or the equipment can be damaged because it is loose on the animal.

In general, several image views are needed to accomplish the exam, which will require constant immobilization of the animal to obtain proper positioning. For instance, Cone Beam Computed Tomography is difficult to implement because it requires that the animal is not only anesthetized but also in the vertical position; thus, an assistant is required, who will be exposed to the radioactive sources. Furthermore, in scintigraphy exams, this person would be in direct contact with either the animal or their secretions. Accordingly, there is the need to create an ergonomic, functional alternative capable of safely enabling imaging exams in several views without the problems mentioned above.

Therefore, the present study aimed to develop and validate an ergonomic and comfortable device for the restraint and immobilization of small animals, in hemodynamically stable conditions and breathing spontaneously, to be used in imaging exams, which provides reduced risk of accidents, easier management, proper isolation, and reduced contamination of both the environment and the assistance team.

## 2. Materials and Methods

The novel Restraining Device for Small Animal Imaging Exams (RDSAIE) was developed in the Experimental Surgery Centre of the Medical School of the Federal University of Rio de Janeiro to facilitate procedures of imaging exams in research projects associated with the Graduate Program in Surgical Science of the Department of Surgery. Its patent has been requested to the National Institute of Industrial Property of Brazil under number BR 20 2014 0250331. In this phase of the project, the innovators are financing its licensing. This study was approved by the University's Institutional Animal Ethics Committee (*Comissão Institucional de Ética no Uso de Animais* (CEUA)), number LABCE02.

### 2.1. Novel Device Description

Initially, a wood prototype was built, which was followed by the construction of the final device in transparent acrylic with a 1.0 cm wall thickness (the cover is 0.5 cm thick). The rectangular containment box has five external parts with the following dimensions: (1) the front side (i.e., the cover), 50 cm height × 22 cm width; (2) the left side, 50 cm height × 15 cm width; (3) the right side, 50 cm height × 16 cm width (with a 0.6 cm wide channel near the front to fit the cover); (4) the back side, 70 cm height × 22 cm width; (5) the bottom, 16 cm on the sides × 22.3 cm in the front and back (with a 0.6 cm wide channel near the front to fit the cover). The upper part of the box has a rectangular opening of 13.3 × 20.3 cm to freely accommodate the head and paws after the animal is positioned and the cover is placed.

**Figure 1:**
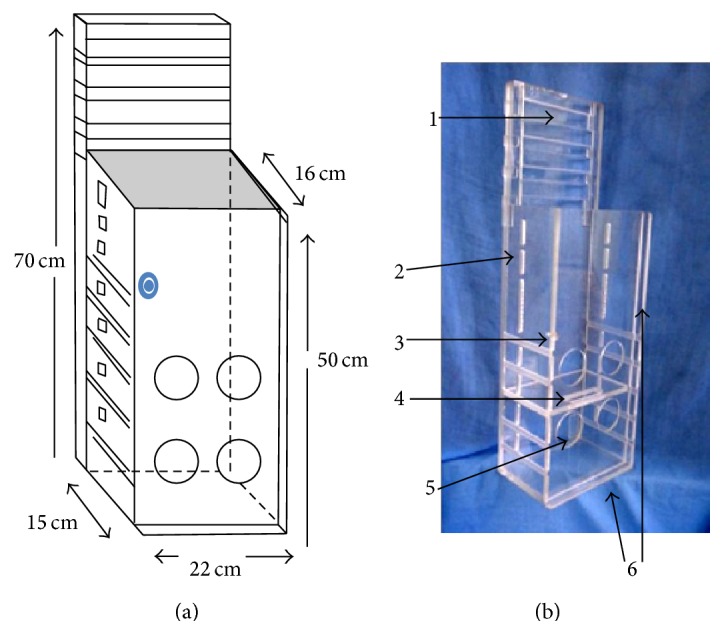
Diagram (a) and photograph (b) of the containment box for small animals. Note in (a) the location of the safety lock on the cover (in blue in the diagram). Note in (b): (1) groove to pass the straps; (2) lateral openings; (3) cover lock; (4) mobile shelf; (5) one of the orifices for the right hind limb; (6) groove for cover fitting.

The cover contains a security lock consisting of an acrylic pin that crosses and couples to an orifice on one side of the box. There is a sliding shelf inside the box with five adjustable levels of height to accommodate the size of the animal. There are seven rectangular, parallel openings on each side of the box that are used to pass Velcro or crepe straps. These straps are passed like a belt over the torso or abdomen and are adjustable to the size of the animal. They surround the posterior part of the box, where they are fixed, thus improving the stabilization. There are also three parallel horizontal channels in the upper extension of the posterior part of the box that allow the positioning of Velcro or crepe straps at the desired height to hold the ears or the frontal region (forehead) of the animal to stabilize the head (Figures 1(a) and 1(b)).

**Figure 2:**
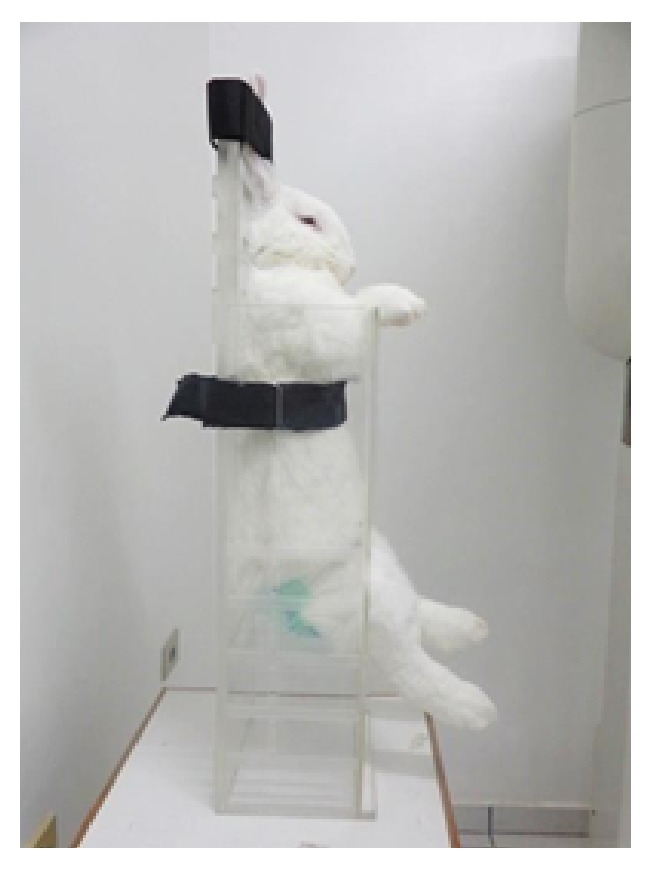
Rabbit placed in the device in the vertical position.

The stabilization of the animal is provided by fitting the shelf at the desired height to accommodate its size. The animal is sited on the shelf with the hind limbs passing through one of the two levels of parallel circular orifices with diameters of 6.3 cm, and the forelimbs are placed on the top of the cover. The tails are comfortably accommodated in a reserved space, without trauma to the animal. The head is fixed with straps and supported by the extension of the posterior wall of the box. The chest or abdomen is also fixed with straps. With the specimen (Figure 2) in the supine position, the box may be positioned for imaging in the vertical or horizontal position. The orifices in the cover and on each side facilitate ventilation inside the box.

### 2.2. Procedures and Imaging Exams

Six New Zealand rabbits (*Oryctolagus cuniculus*) of both genders, weighed between 3 and 4 kg, housed in cages with appropriate environmental conditions, maintained on a circadian cycle and fed with standard industrial rabbit chow and water* ad libitum*, were used in the study. Skull images were obtained after the animals were anesthetized with an intramuscular injection of a solution of 10% ketamine and 2% xylazine hydrochloride at doses of 30 and 10 mg/kg, respectively. Before the imaging tests it was assure that all animal were breathing spontaneously. Magnetic Resonance Imaging (Magneton AvantoA TIM System  1.5 Tesla) and Scintigraphy (Millenium Genie Acquisition GE Release 4.0) with use of the radioisotope technetium^99  MDP^ were performed at the Clementino Fraga Filho University Hospital. The Cone Beam Computed Tomography (Kodak 9500 Cone Beam 3D System) was made at the Dental School of the Federal University of Rio de Janeiro.

**Figure 3:**
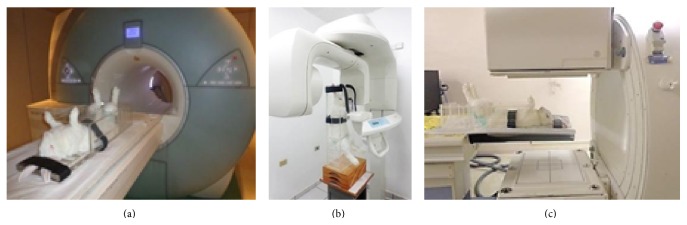
Rabbit positioned for imaging exams. (a) Magnetic Resonance. (b) Cone Beam Computed Tomography. (c) Scintigraphy. Note the stabilization of the body (ear and chest) with straps.

The anesthetized animal was transported to the examination anteroom. Then, a pad was placed on the rabbit within the box. The mobile shelf was adjusted according to the animal's length, and regions of the head and abdomen were restrained (if necessary). Then, the device cover was closed with the hind limbs properly positioned in the orifices of the cover, and the safety lock was engaged. The imaging apparatus was isolated with a plastic or a field and the box containing the animal was then positioned on the equipment (Figure 3).

The test could be interrupted or terminated if the animal did not respond to the anesthetic protocol plan, started to move, or was subjected to other events because the animal remained under supervision with direct visibility through the transparent acrylic box for the entire experiment. The animal was removed from the RDSAIE in the reverse order of the placement, always aiming to provide greater comfort and without inducing pain. In the anteroom, the safety lock was removed from the cover, and the hind limbs were removed from the orifices. Then, the head and abdominal straps were loosened, and the animal was placed in a sealed transfer box in the lateral position for transport to the Experimental Surgery Centre for post- anesthetic observation. At the end of recovery, the animal was sent to the Animal Facility.

## 3. Results and Discussion

Currently, innovation is one of the major assets of a nation. When a new product or piece of equipment is designed that adds functionality and applicability to experimental settings or clinical practice, great value is added to the scientific community. Herein, a containment box that incorporates innovations in veterinary and experimental surgery and that has translational impacts on the surgical and clinical sciences was developed, validated in rabbits, and patented. This device has already been acknowledged by the local scientific community, which has learned of its contribution to facilitate biomedical experiments and data acquisition, thus improving the existing methodological processes. It has the potential to be applied on a larger scale, which shall attract interest for future public-private partnerships, including market or economic sectors associated with manufacturers and distributors of hospital and pet store products.

Once comfortably accommodated in the box, the animal was placed on the test table of the Imaging Centre. Appropriate isolation was observed without any contact with the radiology team. The containment inside the box, use of the pad, and lining of the equipment prevented contamination of the equipment and facilities with organic fluids, especially when radioactive substances were used, such as in scintigraphy. In all equipment used for the imaging exams, the animal remained in the desired position (vertical, horizontal, or decubitus), immobilized with postural stability.

**Figure 4:**
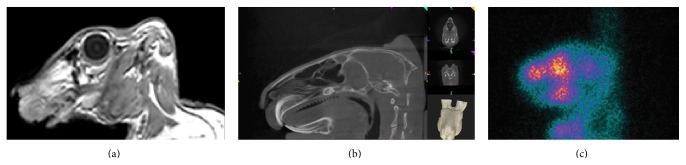
Rabbit skull imaging exams. (a) Magnetic Resonance Imaging. (b) Cone Beam Computed Tomography. (c) Scintigraphy image with radioisotope uptake.

The quality of the images was excellent and showed proper views without any artifacts. Neither the material used to build the box nor the containment straps provided any interference (Figure 4).

The time spent in the examination room was reduced because the animals did not need to be manipulated on the table. The transparent device allowed continuous visual observation of the animals. Undesirable movements or events that would require the administration of more anesthetic or discontinuation of the test were not observed.

The safety and comfort of small animals are extremely important in therapy and for controlled experiments. Thus, the search for a device that allows a postural change without interfering with immobilization, in addition to minimizing handling and direct contact with personnel, is essential for improving the performance of imaging exams in several animal species. Beside these and the ability to immobilize an animal, the RDSAIE has several advantages over other existing devices used in research or veterinary medicine. It is completely constructed of transparent acrylic, which allows visual observation of the animal's behavior; therefore, accidents or compromising the safety of the assistance staff can be avoided. Its sliding removable cover facilitates closing and opening, and the safety pin ensures its fixation. It is adjustable to different animal sizes and accommodates limbs and tail. The pad helps retain excreta that are eliminated during the exam and; in this situation, the box prevents contamination because even the removal of the animal from the radiological centre can be achieved without direct manipulation of the animal.

Among the other containment devices for rabbits, the wood box is the most widely used because it is easy to build. Rothberg [1] patented one in which the rabbit is placed in the ventral decubitus position with limbs tied at length with fixed straps attached to metal knobs. The device has an elongated opening in the top to drain excreta and an internal drawer to contain it. The guillotine-type box for rabbits provides partial containment and is adequate for anesthesia (inhaled or parenteral) and blood collection, which is usually acquired from the marginal ear vein. Thomas [2] conceived one for small animals that exposes the head and immobilizes the neck, the forelimbs in openings in the front wall, and the paws with a strap with a buckle. Fonseca et al. [3] used a wooden box with an anterior cover similar to a guillotine and a base with an orifice through which the animal's head is externalized. The two sides contain the body of the animal, but the posterior part is opened, allowing mobilization of the hind limbs. Polyurethane boxes are also used where the guillotine is fixed with screws. However, these boxes also present the same disadvantages as the wooden box of having a radiopaque structure and not being able to be in the vertical position.

Grbic [4] produced a pet device consisting of suction cups for the legs, hooks, adjustable straps, and Velcro. The retention strips and the suction cups fix the animal's paws on smooth surfaces without impairing the circulation, immobilizing them on the ground during bathing, grooming, nail clipping, and clinical examinations. This device was developed primarily to be used in pet stores and during veterinary clinical examinations on tables or platforms. Unlike the RDSAIE, it does not allow for proper isolation and position changes, in addition to being made of radiopaque materials.

The placement of the animal in a vertical position, which is feasible in the RDSAIE, is rarely found in the descriptions of the literature on this subject. A radiolucent vertical-cephalostat was designed to support and restrain sedated animal's body and head during exposure of images [5]. An immobilization device exists for goats and sheep [6] with a neck restraint with independent regulation to ensure mobilization in a vertical position in a swivel system, and the animal is contained in a lateral structure with support bars and steel cables. It is, therefore, a larger device with screws, rails, and steel, which prevents its use in imaging.

In Brazil [7], a dog and cat restraint has been patented. it has locking systems with anatomic settings comprised of two layered platforms that extend horizontally with lateral projections joined together by screws and protective rounded edges with tracks riveted to the base. Side handles support the nylon straps comprising Velcro and plastic dowels to hold the neck, limbs, shoulders, and back. Nonslip suction cups with screws are used for fixation. Despite being an adjustable platform enabling anesthetic and surgical procedures, the structure is complex and iscomposed of radiopaque material and cannot be used in imaging exams.

Tol and collaborators [8] also built an immobilizer device for animals consisting of a plate with butterfly wing nuts that fix the screws on the front part of the device, which is supported on rubber feet and has an anatomical top opening shaped like a handle for easy transport and handling. It has Velcro straps with adjustable fasteners on the bottom, to accommodate animals of different sizes, fixed by screws and washers. Similar to other animal containment systems, the structure of this device prevents the realization of imaging exams.

Truitt and collaborators [9] developed a passive restraint for small animals that enables transport, comprised of a tube with a protective cone inside, containing an aperture for the tail and another to allow the clearance of urine and excrement. It also has a mechanism that indicates the temperature and a transport tube. Compared to the RDSAIE, it has the disadvantages of being closed tube with a restricted diameter and no transparent capabilities. Custom-made PVC restrainer, which allowed the rabbit to breathe freely without allowing movements of the hind legs, has been used for the longitudinal assessment of osteomyelitis development by molecular imaging of the legs only [10].

CT scans of rabbit's cranium soaked in 10% buffered formalin inside a plastic recipient can be made with the head positioned in a natural way, hugged by wax and supported by wood to keep the acquisition stable [11]. However, a major challenge in clinical-surgical evaluation of small animals is imaging exams, because usually the anesthetized animal is placed on the equipment table either loose or with inadequate straps or lateral restraints. For instance, rabbits have been fixed in the supine position on CT board with thoracoabdominal bandage to reduce motion artifacts [12]. One of the great advantages of the RDSAIE is the possibility of inserting the box in the equipment, enabling decubitus changes during the test without direct contact with the animal and allowing testing in a vertical position, if necessary. When views in the vertical position are indicated, the RDSAIE can be used not only for Cone Beam Computed Tomography, which is specific for dentistry, but also in diagnostic evaluations without difficulty by clinics and hospitals that have conventional X-ray devices.

Cranial neuroimaging exams in animals require head immobilization to avoid the artifacts caused by movement. In a study of fMRI, conscious rabbits were placed in a soft cloth bag that was tied at the neck and tail and fastened to an acrylic cradle with Velcro straps for restraint [13]. An American patent [14] describes a tube for restraining a body portion of the animal that includes a head immobilization mechanism. However, these devices are designed specifically to perform skull studies by nuclear magnetic resonance, which restricts its use for examinations of other parts of the animal. In an elegant study of anesthetized small animal imaging with the use of Cone Beam Micro-CT System [15], perfect axial, sagittal, and coronal views were not obtained due to free animal positioning during exams. The use of restraining devices, especially with the capability of providing orthogonal body positioning, such as in Kim et al. [5], would have improved the beauty of the study results.

No literature was found regarding a device capable of immobilizing small animals with comfort, performing various imaging exams of high quality and without artifacts, in different body positions, especially the vertical, and with safety for both the animal and the professional. It should be noted that the RDSAIE was designed for rabbits, but it can be manufactured in different sizes and scaled to accommodate larger or smaller animals, weighting up to 10 kg. Furthermore, the placement of a handle allows the transport of the animal, by a single person, directly from the animal facility to the imaging exam equipment, dismissing the use of a transport box. A plastic slotted protection cover of the box also avoids animal exposure during transportation.

This device was made specifically for imaging exam that took no longer than one hour, counting from the moment of the anesthesia to the return to the cage in the animal facility. All images performed in the research (Cone Beam Computed Tomography, Magnetic Resonance Imaging, and Scintigraphy) was done with safety in such time interval. It is a useful device that allows visual observation of the animal's behavior after operative procedures in the fields of general surgery, veterinary medicine, and orthopedic and oral surgery, ideal for imaging exams of small animals breathing spontaneously. Of course, longer imaging sessions will require more anesthetics administration and some experiments will require monitoring equipment; however, our goal was to research in animals that were medically capable of undergoing imaging tests without the need for respiratory support or any other monitoring equipment and was not focused on interventional therapies. Nevertheless, for those who wish, it should be easy to install and secure sensors, to monitor the heart rate and pulse oximetry, peripheral venous catheters, or IV lines in one of the paws, for interventional therapies or drug infusions, or to have access to peripheral arteries (i.e., rabbit femoral) for minimally invasive catheter directed therapies such as Transcatheter Intra-arterial Perfusion (TRIP), often done under MRI monitoring. Likewise, it should be easy to apply invasive methods for monitoring life support, such as central vein catheter or endotracheal intubation for inhalant gas anesthetic delivery, increased oxygenation, capnograph assessments and assisted ventilation, since they may be placed with the animal in the supine position inside the box without the cover. After fixing the tubes and wires with tape attached to the box, the cover may be inserted and the desired procedures are performed, even with the animal placed in the vertical position.

We have tested the device with the rabbit inside during the anesthesia recover. It was confirmed that, with the anesthesia protocol utilized, the first sign of arousal was repetitive mandibular movements for about ten minutes, followed by slow body movements. This first sign provides the alert for intervention, if necessary and with plenty of time, before the rabbit starts to move. Even if it does happen, the dimension of the bottom of the box provides sufficient stability to prevent the fall of the animal or the box in any direction. However, there are limitations of the use of the device at the present time. For instance, one should be cautious with animals over four kilograms, because they were not tested.

The prospect of translational studies with the RDSAIE is promising, as it may be adapted for* anima nobile* (e.g., in neonates) by cushioning the areas in contact with the neck and limbs, and by reconfiguring of the orifice positions for the limbs, thus reducing the need for higher level of sedation, the risk of accidents, and the exposure of the assistance staff while performing imaging exams in the radiology unit.

## 4. Conclusions

The Restraining Device for Small Animal Imaging Exams is comfortable, safe, efficient, and ergonomic. It allows the easy placement of animals in different body positions, including the vertical, the maintenance of postural stability and full visibility of the animal behavior. It may be constructed for animals heavier than 4 kg and it is adaptable for translational studies in* anima nobile*.

## Abbreviations

RDSAIE: Restraining Device for Small Animal Imaging Exams.

## Key Points

- The device facilitates conducting small animal imaging exams.
- The device provides restraint with safety and stability.
- The device protects the lab personnel.
- The device facilitates animal positioning in the equipment, including the vertical.
- The device allows full visibility of the animal behavior and proper anesthetic interventions.
